# Irritable Bowel Syndrome and the Menstrual Cycle

**DOI:** 10.7759/cureus.12692

**Published:** 2021-01-14

**Authors:** Girish K Pati, Chinmayee Kar, Jimmy Narayan, Kanishka Uthansingh, Manas Behera, Manoj K Sahu, Debakanta Mishra, Ayaskanta Singh

**Affiliations:** 1 Gastroenterology, Institute of Medical Sciences and SUM Hospital, Bhubaneswar, IND; 2 Obstetrics and Gynecology, SUM Ultimate Medicare, Siksha 'O' Anusandhan University, Bhubaneswar, IND; 3 Gastroenterology, Institute of Medical Sciences and SUM Hospital, SUM Ultimate Medicare, Siksha 'O' Anusundhan University, Bhubaneswar, IND

**Keywords:** constipation, diarrhea, dyspepsia, menstruation

## Abstract

Introduction: Irritable bowel syndrome (IBS) is a common gastrointestinal (GI) disorder. Female patients with bowel disease commonly report worsening of symptoms in the menstrual cycle. However, there is a paucity of data regarding IBS presentations' variation during different phases of the menstrual cycle. The current study aimed to evaluate the same in female IBS patients.

Materials and Methods: Consecutive premenopausal female patients with the diagnosis of IBS attending the outdoor (OPD) of Department of Gastroenterology and Gynaecology; IMS and SUM Hospital, Bhubaneswar, Odisha from June 2019 to December 2019 were included in the study and evaluated by a set of questionnaires regarding various presentations during all the three phases of the menstrual cycle.

Results: Consecutive 102 cases with the diagnosis of IBS were included in the study and evaluated. The mean age of presentation was in mid-30s. Most of the subjects suffered from IBS mixed (IBS-M) type. More patients significantly suffered from constipation (27.27%) during the luteal phase of their menstrual cycles than the menstrual period of their cycles (p - 0.009). More than 50% of all the cases suffered from abdominal bloating during all phases of the menstrual cycle, which was quite disturbing and affected the quality of life. These IBS patients were more symptomatic with more significant limitation of daily activities, suffered from low quality of life, and obtained physician consultation during the menstrual phase as compared to other cycle stages.

Conclusion: Premenopausal female patients with IBS become much more symptomatic during the menstrual phase of the cycle than other stages of the cycle.

## Introduction

Irritable bowel syndrome (IBS) is among the most common gastrointestinal (GI) disorder, affecting around 8.8% of the population [1]. It accounts for significant global morbidity and poor quality of life [1]. Around 4.2%-7.5% of Indian population suffer from IBS [2]. It is usually suspected when someone suffers from abdominal pain and altered bowel habit without any identifiable organic cause. IBS cases amount to around 25%-50% of all GI referral cases as it is common in the community [1-2]. Although the exact aetiology of IBS is yet to be accurately defined but reported, risk factors for IBS were female sex, family history of IBS, personal history of sexual abuse, and presence of anxiety or depression [3].

There are three subtypes of IBS described in the literature; they are diarrhoea-predominant IBS (IBS-D), constipation-predominant IBS (IBS-C), and IBS mixed (IBS-M) having both diarrhea and constipation symptoms intermittently. Although, the pathogenesis of IBS is multifactorial, some studies suggested that sex hormones may play a pivotal role [3]. It has been reported that premenopausal females with IBS often suffered from exacerbating their symptoms during the menstrual period [4-6]. A study by Lewis et al. had revealed that premenopausal females suffer from fewer abdominal bloating bouts than postmenopausal women [7]. The literature also suggests that patients suffer from loose and frequent stools during the menstrual period than the luteal phase of the menstrual cycle [8]. This type of difference in bowel pattern may be related to female sex hormones such as progesterone and estrogen, whose blood concentration remains relatively higher during the luteal phase than the menstrual phase. These progesterone and estrogen hormones may play a role in the GI transit time [9]. On the contrary, some studies suggest that rectal motility and sensitivity are usually not altered in healthy women during the menstrual period [8, 10].

The study aimed to evaluate the bowel disturbances in premenopausal females with relation to their menstrual cycle and associated dyspeptic symptoms in these female patients.

## Materials and methods

This study was a single centered, cross-sectional, open labeled, nonblinded, noninterventional cohort study. Consecutive premenopausal females diagnosed with IBS attending the Department of Gastroenterology and Gynecology; IMS and SUM Hospital, Bhubaneswar, Odisha from June 2019 to December 2019 were included in the study and evaluated by a set of questionnaire based on the bowel and dyspeptic symptoms. This questionnaire was nonvalidated and self-designed. 

The inclusion criteria of the study were premenopausal females, age ≥ 18 years, and diagnosed case of IBS.

The exclusion criteria are males, postmenopausal females, females with primary amenorrhea, females < 18 years, pregnant females, those with organic bowel diseases such as inflammatory bowel disease (IBD), ischemic colitis, radiation colitis, microscopic colitis, infective colitis, eosinophilic colitis, colorectal malignancy, tubercular colitis, females with red flag signs such as loss of weight, anaemia, history of intermittent fever, nocturnal diarrhoea, history of lower gastrointestinal (LGI) bleeding, and refusal to participate in the study.

IBS patients were selected as per Rome IV criteria [1]. Rome IV criteria state that a case of IBS is diagnosed when there is the presence of recurrent abdominal pain for at least one day a week during the last three months with symptom onset at least six months beforehand or more and associated with two or more of the following: (i) the pain is related to defecation, and/or (ii) onset is associated with a change in stool frequency; and/or (iii) onset is associated with a difference in stool quality.

All the selected patients underwent a full colonoscopy and gastroduodenoscopy with segmental biopsies to rule out any organic pathology. These patients also had an ultrasonography study of abdomen and pelvis done to rule out any abdominal and genitourinary abnormalities. All the cases were assessed about the changes in their bowel symptoms during the three phases of their menstrual cycles, i.e., the menstrual phase (1 to 7 days of starting of menstruation), follicular phase (8 to 14 days after the beginning of menstruation), and luteal phase (15 to 28 days after the onset of menstrual bleeding).

However, as the sensitivity of Manning criteria for the diagnosis of IBS was relatively high compared to other diagnostic criteria in India [11], the questionnaire had included variables from the Manning criteria. As IBS and functional dyspepsia (FD) may coexist as both of them have common pathophysiological mechanisms [12], hence dyspepsia was also evaluated. Each of the participants gave informed written consent before inclusion into the study protocol.

Statistical analysis

The SPSS software version 21 (SPSS for Windows, version 21.0, SPSS Inc., Chicago, USA) was used to perform all statistical analyses. The results were expressed as mean ± standard deviation (SD) or frequency in percentage. Normally distributed quantitative and categorical variables were compared using Student's t-test and Chi-square test, respectively.

## Results

A total of 134 consecutive female patients were screened and after the exclusion, 102 premenopausal females with a diagnosis of IBS were included in the study and evaluated. The mean age of presentation was 34.13 ± 6.86 years. The majority (44.11%) of the subjects were aged 26-35 years, 12.74 % cases were aged 18-25 years, and the rest (43.13%) were aged in the range of 36-45 years. Mean duration of the disease was long (mean of 10 years). The mean hemoglobin (Hb) of the cases was 11.51 ± 2.23 g%. In this study, 40%, 55.55%, and 49% of cases suffered from poor appetite during menstrual, follicular, and luteal phases of their menstrual cycles, respectively. Table 1 describes all the baseline findings.

Table 2 narrates the frequency of IBS symptoms during various stages of the menstrual cycle. It was noted that patients had a significant association with diarrhoea during the menstrual phase compared to other stages, whereas constipation was more common in the luteal grade. Table 3 shows dyspeptic symptoms in all the cases during different phases of the menstrual cycle. More than 50% of the subjects suffered from abdominal bloating during all the menstrual cycle phases, which was quite disturbing and affected the quality of life. Comparative analysis of all the cases during different phases of the menstrual cycle was described in Table 4. Comparative analysis of dyspeptic symptoms in all the cases during different phases of the menstrual cycle was described in Table 5.

**Table 1:** Baseline parameters in all the studied patients.

Findings in all the cases (N = 102)	Values
Mean age of presentation	34.13 ± 6.86 years
Mean hemoglobin	11.51 ± 2.23 g%
Weekly defecation frequency	11.12 ± 6.06 times
Daily defecation frequency	1.58 ± 0.86 times
Abdominal fullness/bloating	63.72% cases
Post defecation relief from pain abdomen	80.39% cases
Passage of mucoid stool	36.27% cases
Urgency of stool	61.76% cases
Feeling of Incomplete evacuation	67.64% cases
Straining during defecation	60.78% cases
Mean disease duration	10.47 ± 11.91 years
Relief of pain following flatus passage	80.39% cases
Increased defecation frequency at the onset of pain abdomen	8.82% cases
Defecation frequency > 3 times / day	8.82% cases
Cases seeking physicians' consultations	88.23% cases
Associated post prandial fullness	86.27% cases
Associated epigastric pain	61.73% cases
Associated early satiety	34.31% cases
Associated epigastric burning sensation	52.94% cases
Symptoms interfering regular daily activities	95.09% cases

**Table 2:** Bowel symptoms during different phases of menstrual cycle.

Findings (% Cases)	Menstrual	Follicular	Leuteal
Constipation	0	14.81	27.27
Diarrhea	15	11.11	7.25
Abdominal bloating	65	55.55	65.45
Post defecation pain relief	90	70.37	81.81
Passage of mucus	30	44.44	34.54
Urgency for defecation	70	59.25	60
Feeling of incomplete defecation	55	74	69
Pain relief by passage of flatus	90	70.37	81.81
Increased defecation frequency at the onset of abdominal pain	20	18.51	18.18
More than three times daily defecation	10	7.4	9
Physician consultation taken	100	85.18	85.45
Interference in daily activities	100	92.59	94.54

**Table 3:** Dyspeptic symptoms during different phases of menstrual cycles.

Findings (%)	Menstrual	Follicular	Luteal
Post prandial fullness	95	92.59	80
Early satiety	30	37	34.54
Epigastric pain	75	70.37	52.72
Burning epigastrium	50	62.96	49

**Table 4:** Comparative analysis of different dyspeptic symptoms in all the cases during different phases of menstrual cycles.

Findings (%)	p-value in between menstrual and follicular phase	p-value in between follicular and luteal phase	p-value in between luteal and menstrual phase
Post prandial fullness	0.68	0.16	0.11
Early satiety	0.61	0.78	0.74
Epigastric pain	0.7	0.12	0.07
Burning epigastrium	0.41	0.26	0.93

**Table 5:** Comparative analysis of different findings during different phases of menstrual cycle.

Findings (%)	p-value in between menstrual and follicular phase	p-value in between follicular and luteal phase	p-value in between luteal and menstrual phase
Constipation	0.08	0.18	0.009
Diarrhea	0.68	0.53	0.28
Abdominal bloating	0.49	0.38	1
Post defecation pain relief	0.09	0.26	0.35
Passage of mucus	0.32	0.37	0.74
Urgency for defecation	0.43	0.93	0.42
Feeling of incomplete defecation	0.17	0.64	0.26
Pain relief by passage of flatus	0.09	0.26	0.35
Increased defecation frequency at the onset of abdominal pain	0.86	1	0.84
More than three times daily defecation	0.71	0.75	0.89
Physician consultation taken	0.07	1	0.06
Interference in daily activities	0.19	0.59	0.26

A relatively more number of cases had post defecation pain relief during the cycle's menstrual phase than other phases of the cycle. More cases passed mucus in stool during the follicular phase compared to other stages of the cycle. The urgency for defecation was maximally complained during the menstrual phase compared to other phases of the cycle. Patients suffered less commonly from incomplete evacuation during the menstrual phase than other stages of the cycle. Relief from abdominal pain following the passage of flatus was maximally observed during the menstrual phase of the cycle compared to other phases. All the cases had a low quality of life, limited daily activities, and obtained physician consultation more during the cycle's menstrual phase. Although premenopausal patients with IBS become much more symptomatic during the menstrual phase of the cycle than other phases of the cycle, these do not attend statistical significance (Table 4).

## Discussion

This study evaluated various bowel symptoms during these three periods of menstrual cycles. Although most of the cases suffered from IBS MIXED (IBS-M) type in this study, we found that 27.27% of the patients suffered from constipation predominantly during the luteal period of their menstrual cycles. In contrast, no one suffered from constipation primarily during the menstrual period of their cycles, which was statistically significant (p - 0.009). Published literature reported that sex hormone receptors are present along the GI tract, affecting GI motility during the menstrual cycle [9]. Previous studies have suggested that constipation during the menstrual cycle's luteal phase may be related to high progesterone and oestradiol levels during this period [9]. Literature indicates that female sex hormones might modulate response to stress, gut motility, and visceral pain perception by influencing the gut-brain axis and interacting with emotional and neuromodulatory system [13]. Estrogen and progesterone usually inhibit smooth muscle contraction; therefore, they may affect gastrointestinal (GI) motility. It was also highlighted that progesterone modulates the colonic 5-hydroxytryptamine (5-HT) activity, which is known to control peristalsis. Wald et al. reported that significantly longer gut transit time occurs during the luteal phase than the follicular phase [9]. It was not surprising that some studies have shown an increased gut transit time during the luteal phase [9]. However, few conflicting studies have shown no changes in gut-transit time [14]. The contradictory results can be due to the differences in study populations, study designs, and the markers used to measure GI transit time.

Out of all cases, 15% of patients suffered from diarrhea predominantly during the menstrual phase of their cycle. A relatively less number of cases suffered from diarrhea, mostly during the follicular and luteal phases of their cycle. The following reasons can explain this: the menstrual period starts with an increase in uterine prostaglandins (PGs), particularly PGF2a and prostacyclin, which stimulates GI motility, thereby increasing occurrence of diarrhea [15]. The estrogen level has two peaks; one peak during the mid follicular phase and another peak during the early luteal phase [16]. Estrogen level drops down following ovulation, followed by the rise in both estrogen and progesterone levels during the early luteal phase [16]. The falling level of both hormones in the late luteal stage leads to menses. As progesterone leads to decreased GI motility; its reduced level during the menstrual period leads to increased diarrhea. Estrogen has a positive effect on GI motility through estrogen receptor-dependent and independent mechanisms [17]. Estrogen receptor-independent mechanisms include direct activation of potassium channels and direct inhibition of voltage-dependent calcium channels. On the contrary, progesterone acts via the progesterone receptors regulating intracellular G proteins which mediate smooth muscle relaxation.

These IBS patients are more symptomatic with more significant daily activities, suffered from low quality of life, and obtained more physician consultations during the menstrual phase than other phases of the cycle. However, these parameters did not achieve statistical significance. A meta-analysis reported that the menstrual cycle significantly affects bowel habits and may increase diarrhea, bloating, and abdominal pain [18]. However, several other studies reported minimal effects of the menstrual cycle on orocecal, colonic, or whole gut transit [8]. Possible explanations for this type of discrepancy may be due to different methods used to measure GI transit (hydrogen breath test, scintigraphy, and radio-opaque markers), sample size, heterogeneous patient population, and non-validation of the cycle phase. Other studies also supported that menstruation was associated with significant worsening of IBS symptoms [4-6]. There was worsening of abdominal pain and bloating and more frequent bowel movements during menstruation [4-6]. These studies also reported firmer stool consistency during the luteal phase [4-6]. The same findings were observed in the present study. It has been reported that females with IBS suffered inappropriate somatic and visceral hypersensitivity, which might be linked temporally with the dynamic decrease in the levels of ovarian hormones during menstruation [19]. Houghton LA et al. reported that rectal sensitivity, which involves self-reported discomfort, urgency, and desire to defecate in response to balloon insufflations on barostat examination, varies significantly menstrual cycle in women with IBS, in contrast to healthy controls [20].

 The prevalence of IBS in females occurs most commonly from the late teen to the mid-forties. After the mid-fifth decade, IBS incidence in women decreases with increasing age and may have a secondary peak around 70 years of age or, beyond. In contrast, the prevalence of IBS among men remains constant from third to eight decades of age. Estrogen, one of the female sex hormone, modulates pain perception by involving the afferent sensory system, opioidergic and serotonergic systems, and stress responses in variable ways [21-23]. Estrogen modulates pain perception in the neurons by affecting the glutamatergic activity and increasing neurotrophins synthesis [22-23]. Estrogen also modulates opioid receptor-mediated neurotransmission in the thalamus, nucleus accumbens, and amygdala [22]. Besides, estrogen enhances serotonergic response in the central nervous system (CNS) by increasing synthesis, decreasing uptake and reducing degradation, and uplifting post-synaptic responsiveness [23]. Estrogen may modulate pain perception by affecting the autonomic nervous system (ANS) and the hypothalamic-pituitary axis [24]. Reported studies had demonstrated an increase in sympathetic tone in the mid to late luteal phase when serum estrogen and progesterone were at their highest levels [24]. Progesterone and its metabolites may modulate afferent sensory pathways, which presumed to be central inhibitory receptors in the CNS [25], through their effect on the c-aminobutyric acidergic system. These ovarian hormones can also influence visceral sensitivity and inflammation through serotonergic pathways and regulate mast cell function and response to stress. During the late luteal phase, decreased serum estrogen and progesterone were usually associated with increased colonic 5-hydroxytryptamine-3 (5-HT) receptor expression, leading to increased gastrointestinal symptoms visceral sensitivity [26].

Additionally, estrogen may cause mast cell degranulation and inflammatory mediators' release, increasing visceral sensitivity [27]. Even, estrogen can modulate cortisol receptors within the enteric neuron at a time of stress, with the resultant increase in visceral sensitivity [27]. Also, ovarian hormones may modulate visceral sensitivity by variably affecting nuclear kappa 1 (NK1) receptors in the colon [28]. Thus, estrogen and progesterone levels may lead to either proceptive or antinociceptive effects, based on their modulated pathways, serum concentrations, phase of the menstrual cycle, and their net effect. Jamieson et al. suggested that dysmenorrhea was relatively more common in females with IBS than non-IBS cases [29]. Although the pain was almost always present, it was difficult to differentiate whether the pain was from the uterus or the irritable bowel. Altman et al. reported that higher pain score was more commonly found during the late luteal and menstrual phases of the cycles in cases with concomitant IBS and dysmenorrhea than IBS subjects alone [30].

 IBS and functional dyspepsia (FD) may frequently coexist. In a multi-centric Indian study {Multi-centric Indian IBS study (MIIBS)}, it was reported that the Manning criteria had the highest sensitivity (91%), to diagnose IBS compared to other diagnostic criteria such as Asian criteria (74.5%), Rome I (68%), Rome III (52.5%) and Rome II (40%) [11]. Prior studies reported that around 56% to 87% of IBS cases might suffer from FD [12]. In this study, 30 to 95% of patients suffered from different dyspeptic symptoms during various phases of the menstrual cycles. This study observed that postprandial fullness followed by pain in epigastrium occurred in many cases, irrespective of the menstrual cycle phases. IBS and functional dyspepsia (FD) may coexist as both of them have common pathophysiological mechanisms, like visceral hypersensitivity, abnormal sensory perception, gastrointestinal (GI) dysmotility and putative psychosis [12]. 

 Thus, it is likely that female sex hormones have a more prominent role in modulating IBS symptomatology in females. It was also supported that IBS symptoms may improve following oophorectomy and by using GnRH agonists [21]. Hormonal therapy may help female IBS patients, but further extensive prospective studies are required for validation.

There are a few limitations in this study. Objective pain score for IBS is not done in this study. The prevalence of any psychosomatic symptoms is not studied. The measurement of sex hormones in different phases of the menstrual cycle and its correlation with bowel symptoms can throw additional light on the pathogenesis.

As most of the published studies have several limitations like retrospective design, small sample sizes, non-standardized self-report of symptoms and sizeable inter-individual variability, and without any assessment about levels of sex hormones during different phases of the menstrual cycle, research should focus on the prospective recording of symptoms and appropriate examination of sex hormones during various stages of the menstrual cycle. Also, the levels of sex hormones should be assessed not only from serum but also from urine. An essential next step is to determine whether sex hormones can play a role in the current therapeutic approaches for IBS in females.

## Conclusions

Premenopausal female patients with IBS may suffer from fluctuating symptoms during different phases of the menstrual cycle, which correlates with the fluctuations in their sex hormones' level. However, further prospective studies are required before sex hormones can be considered as therapeutic interventions for IBS. Categorization and selection of patients on IBS subtypes may also be needed in these studies.
